# Optimizing reproducibility of operant testing through reinforcer standardization: identification of key nutritional constituents determining reward strength in touchscreens

**DOI:** 10.1186/s13041-017-0312-0

**Published:** 2017-07-17

**Authors:** Eun Woo Kim, Benjamin U. Phillips, Christopher J. Heath, So Yeon Cho, Hyunjeong Kim, Jemeen Sreedharan, Ho-Taek Song, Jong Eun Lee, Timothy J. Bussey, Chul Hoon Kim, Eosu Kim, Lisa M. Saksida

**Affiliations:** 1 Department of Psychiatry, Institute of Behavioral Science in Medicine, BK21 Plus Project for Medical Sciences, Severance Biomedical Science Institute, Yonsei University College of Medicine, 50-1 Yonsei-ro, Seoul, 03722 Republic of Korea; 2 Department of Psychology and MRC/Wellcome Trust Behavioural and Clinical Neuroscience Institute, University of Cambridge, Downing Street, Cambridge, CB2 3EB UK; 3 School of Life, Health and Chemical Sciences, The Open University, Walton Hall, Milton Keynes, MK7 6AA UK; 4 The Babraham Institute, Cambridge, CB22 3AT UK; 5 Maurice Wohl Clinical Neuroscience Institute, Institute of Psychiatry, Psychology and Neuroscience, King’s College London, 5 Cutcombe Road, London, SE5 9RT UK; 6 Department of Radiology, Severance Biomedical Science Institute, Yonsei University College of Medicine, 50-1 Yonsei-ro, Seoul, 03722 Republic of Korea; 7 Department of Anatomy, BK21 Plus Project for Medical Sciences, Severance Biomedical Science Institute, Yonsei University College of Medicine, 50-1 Yonsei-ro, Seoul, 03722 Republic of Korea; 8 Molecular Medicine Research Laboratories, Robarts Research Institute & Department of Physiology and Pharmacology, Schulich School of Medicine & Dentistry, The Brain and Mind Institute, Western University, London, ON Canada; 9 Department of Pharmacology, BK21 Plus Project for Medical Sciences, Severance Biomedical Science Institute, Yonsei University College of Medicine, 50-1 Yonsei-ro, Seoul, 03722 Republic of Korea

**Keywords:** Reinforcer, Operant behavior, Calories, Touchscreen chamber, Standardization

## Abstract

Reliable and reproducible assessment of animal learning and behavior is a central aim of basic and translational neuroscience research. Recent developments in automated operant chamber technology have led to the possibility of universal standard protocols, in addition to increased translational potential, reliability and accuracy. However, the impact of regional and national differences in the supplies of available reinforcers in this system on behavioural performance and inter-laboratory variability is an unknown and at present uncontrolled variable. Therefore, we aimed to identify which constituent(s) of the reward determines reinforcer strength to enable improved standardization of this parameter across laboratories. Male C57BL/6 mice were examined in the touchscreen-based fixed ratio (FR) and progressive ratio (PR) schedules, reinforced with different kinds of milk-based reinforcers to directly compare the incentive values of plain milk (PM, high-calorie: high-fat/low-sugar), strawberry-flavored milk (SM, high-calorie: low-fat/high-sugar), and semi-skimmed low-fat milk (LM, low-calorie: low-fat/low-sugar) on the basis of differences in caloric content, sugar/fat content, and flavor. Use of a higher caloric content reward was effective in increasing operant training acquisition rate. Total trial number completed in FR and breakpoint in PR were higher using the two isocaloric milk products (PM and SM) than the lower caloric LM, with comparable outcomes between PM and SM conditions, suggesting that total caloric content determines reward strength. Analysis of within-session changes in response rate revealed that overall outputs in FR and PR primarily depend on the response rate at the initial phase of a session, which itself was dependent on reinforcer caloric content. Interestingly, the rate of satiation, indicated by decay in response rate within a FR session, was highest when reinforced with SM, suggesting a rapid satiating effect of sugar. The key contribution of reward caloric content to operant performance was confirmed in a multi-laboratory study using the touchscreen 5-choice serial reaction time task (5-CSRTT) reinforced by two isocaloric milk-based liquid rewards with different countries of origin, which yielded consistent performance parameters across sites. Our results indicate that milk-based liquid reinforcer standardization can be facilitated by matching caloric content across laboratories despite regional or national differences in other non-caloric aspects of the reinforcers.

**Electronic supplementary material:**

The online version of this article (doi:10.1186/s13041-017-0312-0) contains supplementary material, which is available to authorized users.

## Introduction

Reliable and reproducible assessment of animal learning and behavior is a central aim of basic and translational neuroscience research [1, 2]. Recent developments in operant chamber technology such as the touchscreen testing system have resulted in the increasing use of these automated systems with computerized data collection and analysis facilities – in addition to higher translational potential and increased reliability and accuracy, this has also led to the possibility of universal standard protocols across departments in institutions across the globe [3–7].

Whilst apparatus, stimuli, behavioral protocol and software homogeneity have been achieved, one as yet uncontrolled variable that could hinder complete standardization concerns the reinforcers used to support animal behavior. Training and testing experimental animals in automated operant systems typically relies on appetitive motivation by provision of a food reward upon the emission of specific behavioral responses in the context of mild food restriction. Moreover, previous studies have demonstrated that operant learning and task performance in rodents can be crucially affected by the attributes of the reinforcer selected such as taste, state (liquid versus solid), nutritional value, and palatability [8, 9].

While solid rewards typically in the form of 20 or 40 mg pellets can be used to reinforce rodent behaviour, the use of liquid reinforcement in mice is of particular benefit, as it minimises the impact of potential confounds such as dry mouth or an inability to chew solid reinforcers [3] which can occur in genetically modified models. In addition, liquid reinforcement may be more effective than solid reward pellets [8], and a recent study from our group has indicated that among liquid reinforcers, milk-based solutions are superior to sweetened non-milk liquids such as super-saccharin solution in the touchscreen system [9].

Accordingly, several commercial milk-based products including dilute sweetened milk and various flavours of milkshake have been used in operant behavioral studies [8, 10–13]. Given the diversity of liquid reinforcers used, it is highly likely that reinforcer strength differs between studies. This could impact operant output and in turn alter key performance parameters such as the number of training sessions required to reach a standardized performance criterion or the maximum performance level of a given strain of mice on a particular task. Moreover, the availability of a specific product to be used as a reinforcer cannot always be guaranteed for laboratories in different countries due to regional and national variations in suppliers. This lack of consistency in reinforcer may contribute to inter-laboratory variation, make comparisons between studies difficult and potentially compromise multi-site investigations. Therefore, it is critical to identify which factor(s) determine the incentive value of liquid reinforcement used in operant procedures in order to facilitate standardization of liquid reward between laboratories using locally available products.

In this study, we assessed the performance of C57BL/6 mice on touchscreen-based, fixed-ratio (FR) and progressive-ratio (PR) schedules using a series of nutritionally distinct reinforcers. These ratio schedules have been used to measure the reinforcing potential of rewards [14] as well as the motivational levels of laboratory animals in the touchscreen apparatus [10]. We also conducted a dual site study in which we compared the behavioral outcomes in the 5-CSRTT [5] reinforced with isocaloric milk-based products with different countries of origin. Here we report that the caloric content per unit of reinforcer, rather than the fat content, sugar content or flavor, should be made equivalent between institutions to ensure standardized reinforcer value and operant performance.

## Results

### FR response is dependent on reinforcer caloric content

We compared three milk products that have distinct nutritional profiles (Table 1); The semi-skimmed low-fat milk (LM) has 40 kcal/100 mL (low-calorie) and both strawberry-favored milk (SM) and plain milk (PM) have 65 kcal/100 mL (high-calorie). SM and PM, though equivalent in calories (isocaloric), are different in their composition; SM derives 62% of its total calories from sugar (high-sugar) whereas the fat content of PM constitutes 49% of the total caloric content of this liquid (high-fat). Both SM and PM have a 15–18% amino acid contribution to their total caloric content.

**Table 1:** Nutritional features of milk reinforcers used and purpose of selection

per 100 mL	Low-Fat Plain Milk (LM)	Strawberry Milk (SM)	Plain White Milk (PM)
Calories (kcal)	40	65	65
Total Carbohydrates(g)	4.5	10.5	4.5
Sugars (g)	4.5	10	4.5
Protein (g)	3	2.5	3
Total Fat (g)	1	1.5	4
Saturated Fat (g)	0.5	1	2.5
Cholesterol (mg)	2.5	5	10
Sodium (mg)	50	45	50
Intent of contrast			
Caloric content	low	high	high
Fat/sugar content		high-sugar	high-fat
Flavor		strawberry	plain

**Fig. 1:**
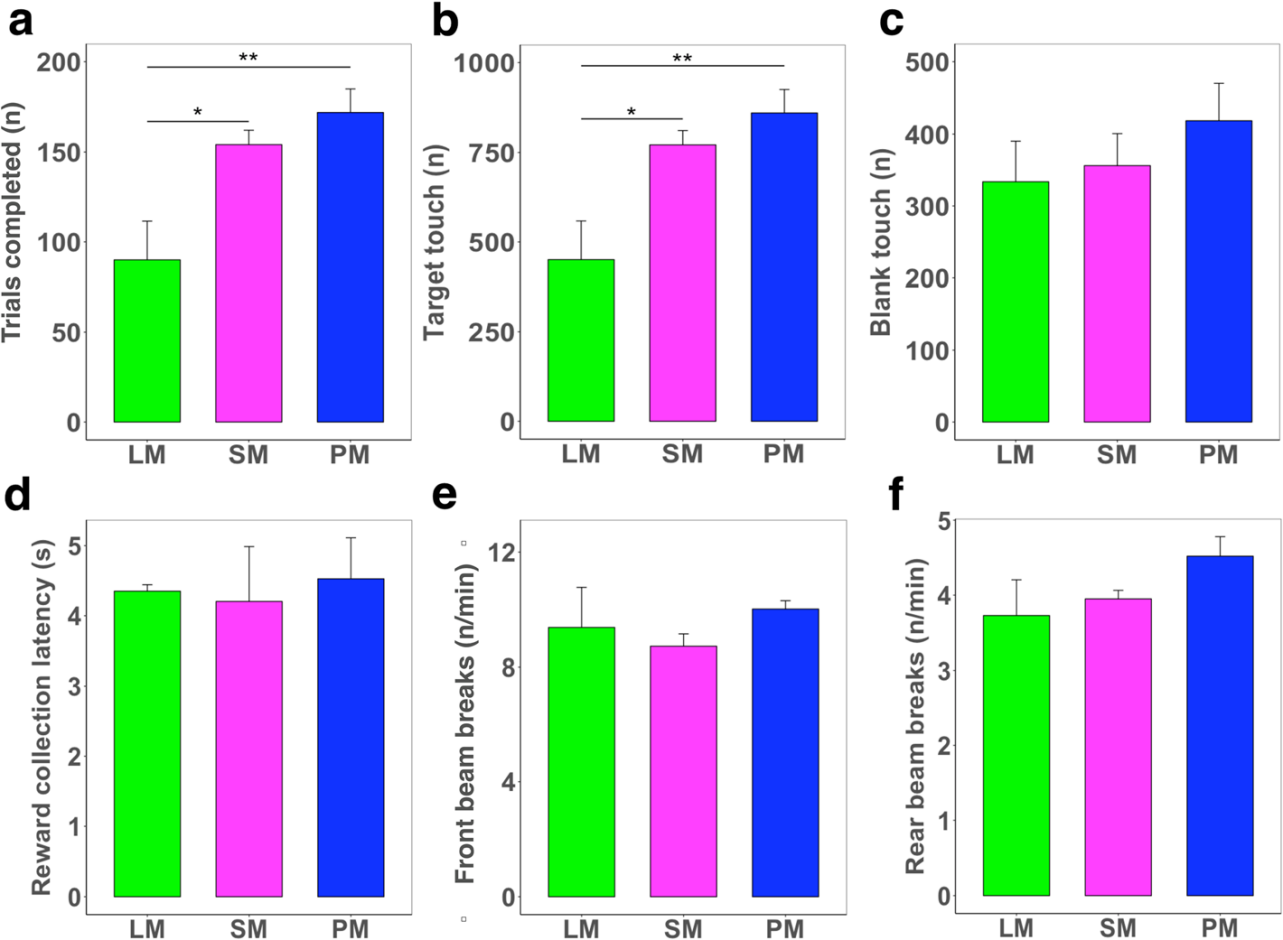
Performance of male C57BL/6 mice in the unrestricted FR5 schedule **a**. The total number of trials completed (one-way ANOVA; F = 7.48, df = 2,11, *p* < 0.01). **b**. Target touches, the total number of responses to the correct touchscreen response location (one-way ANOVA; F = 7.47, df = 2,11, *p* < 0.01). **c**. Blank touches, responses to incorrect touchscreen locations (n.s.) **d**. Reward collection latency, the time between reward delivery to the magazine and reward collection by the animal (n.s.). **e-f**. Rate of IR beam breaks in the front and rear areas of the touchscreen chamber (n.s.). LM, low-fat milk; SM, strawberry-flavored milk; PM, plain white milk. Note that SM and PM are isocaloric (65 kcal/100 mL) but have different sugar and fat compositions. LM is low calorie (40 kcal/100 mL). *n* = 3–7 per group. n.s., not significant. *p < 0.05; **p < 0.01 in Tukey’s post hoc comparison

First, we conducted an unrestricted FR5 schedule, in which mice can emit an unlimited number of responses within 60 min. FR5 requires that five consecutive touchscreen responses be emitted to earn a single reward. As the FR schedule requires invariant effort expenditure to earn each reward, it can indicate not only levels of motivation for food ingestion but also the threshold of satiety for individual animals. We found that the two reinforcers with higher caloric content (SM and PM) induced significantly higher numbers of trials completed and total touches to target than the lower calorie reinforcer (LM), yet comparable outcomes between the two (Fig. 1a and b), indicating that FR performance generally depends on reinforcer total caloric content rather than on fat or sugar content in isolation or on flavor. The number of total touches to incorrect touchscreen response locations (blank touches) did not differ between groups (Fig. 1c). Reward collection latency, and the rate of infrared (IR) beam breaks were also not different between groups (Fig. 1d-f), indicating no effects of nutritional contents on general locomotor activity of animals during the task performance. While conducting FR and PR schedules, average weights of animals were comparable between groups (Additional file 1: Figure 1Sa).

### PR response is dependent on reinforcer caloric content

**Fig. 2:**
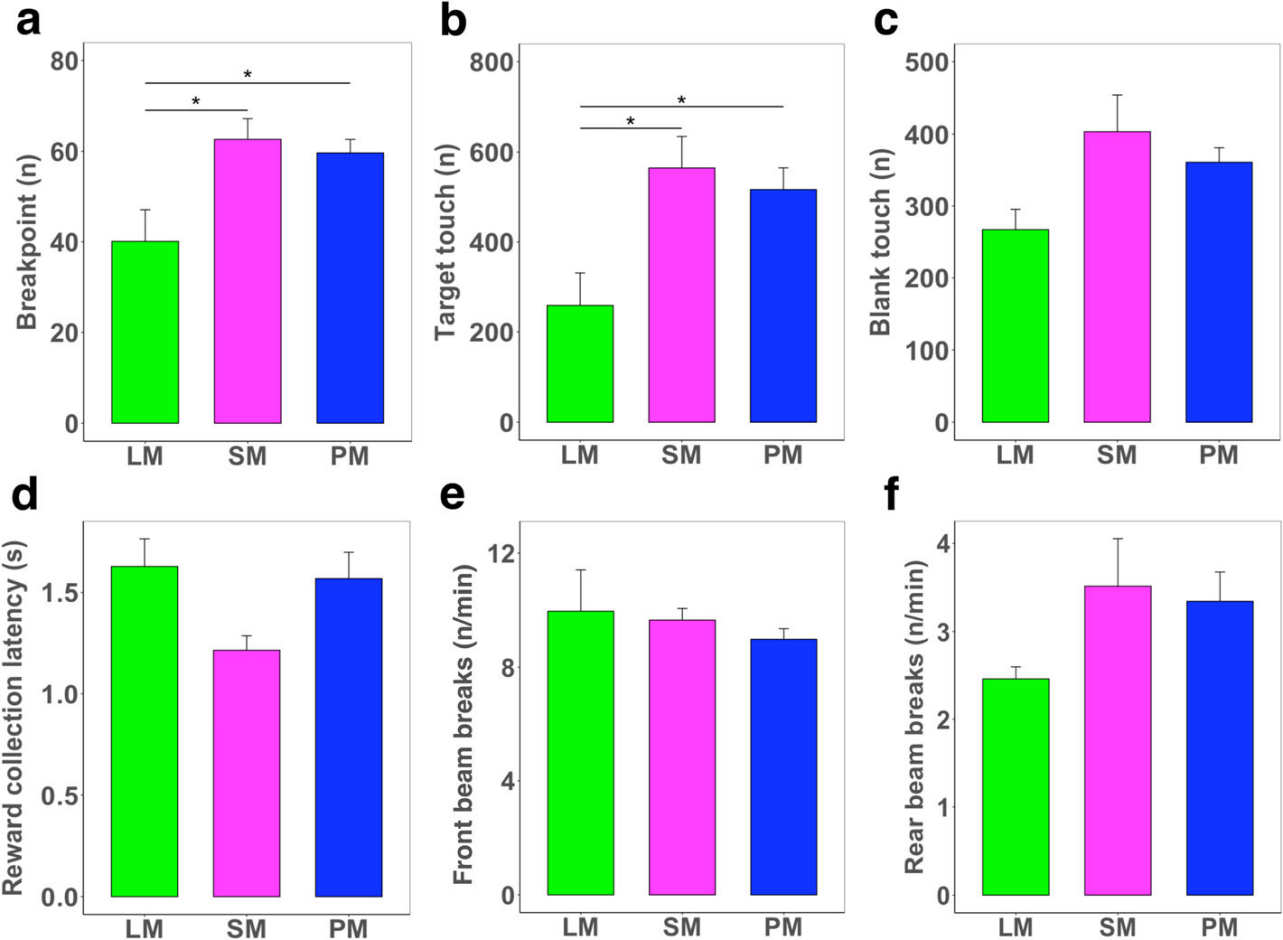
Performance of male C57BL/6 mice in the PR4 schedule **a**. Breakpoint (one-way ANOVA, F = 6.24, df = 2,11, *p* = 0.015). **b**. Target touches (one-way ANOVA, F = 6.24, df = 2,11, *p* = 0.015). **c**. Blank touches (one-way ANOVA, F = 3.44, df = 2,11, *p* = 0.07) **d**. Reward collection latency (n.s.). **e-f**. Rate of IR beam breaks in the front and rear areas of the touchscreen chamber (n.s.). LM, low-fat milk; SM, strawberry-flavored milk; PM, plain white milk. *n* = 3–7 per group. n.s., not significant. *p < 0.05 in Tukey’s post hoc comparison

Next, we examined the performance of mice in the PR4 schedule to identify differences in the incentive value of the three reinforcers. This is indicated by measuring animals’ effortful food-seeking behaviors as in this schedule the operant response requirement increases predictably (by an additional 4 responses) with every subsequent trial [15]. Similar to the findings from the FR schedule, both the breakpoint (the number of touchscreen responses emitted on the last successfully completed trial which is used as an indicator of motivation or incentive value of reward; Fig. 2a) and the total number of target touches differed according to the total reinforcer caloric content (Fig. 2b). Blank touches, reward collection latency, and the IR beam break rate did not differ significantly between groups (Fig. 2c-f).

### Initial response rate is dependent on reinforcer caloric content as a determinant of overall output in the FR and PR schedules

**Fig. 3:**
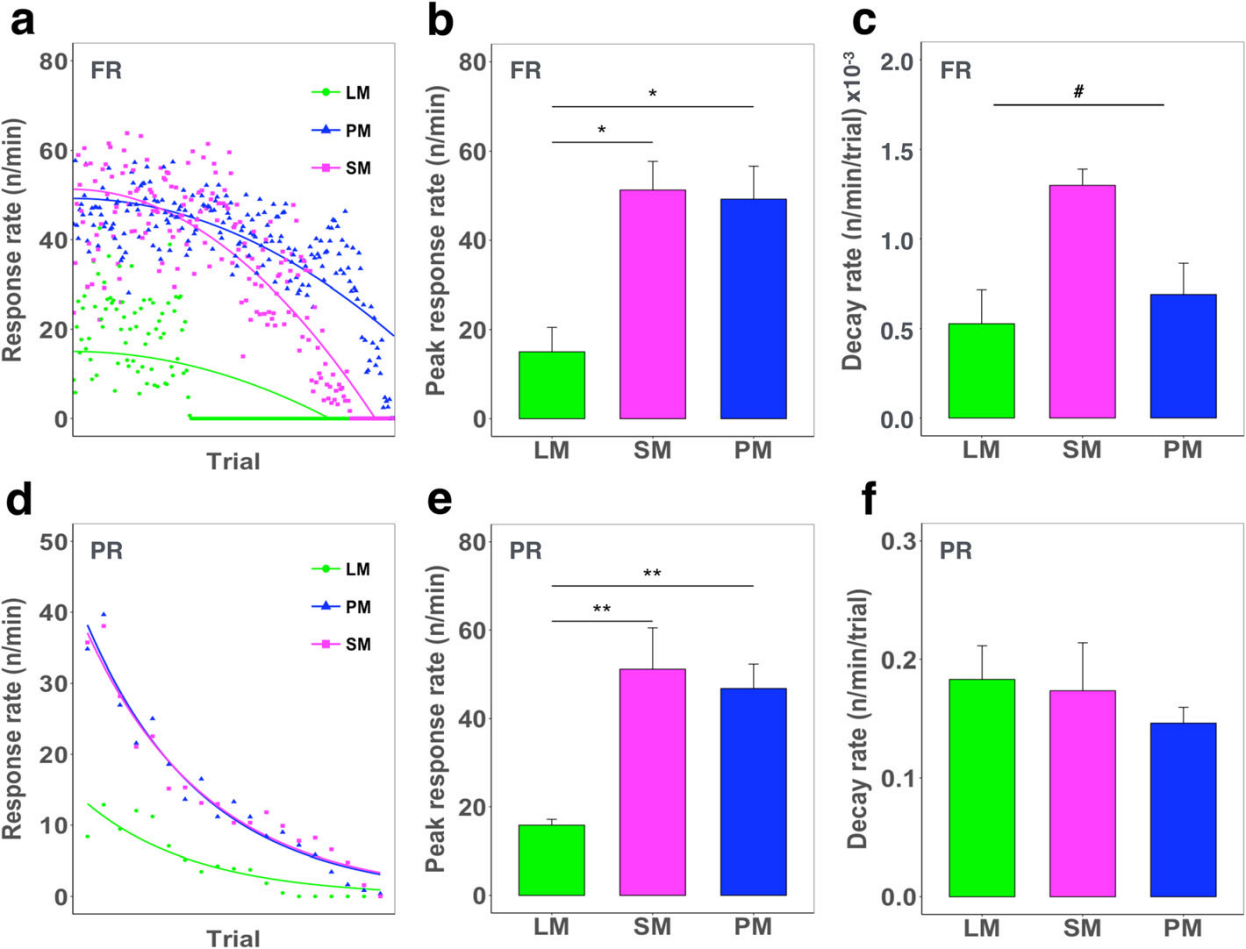
Within-session response rate analysis in the FR5 and PR4 schedules. **a**. Group mean responses per minute in each trial under an unrestricted FR5 schedule. **b**. Predicted peak response rate in FR (one-way ANOVA, F = 5.36, df = 2, 11, *p* < 0.05). Post-hoc testing revealed that PM and SM supported a significantly higher predicted peak response rate compared to LM (*p* < 0.05). **c**. Analysis of response decay rate in FR (one-way ANOVA, F = 4.53, df = 2, 11, *p* < 0.05). Post-hoc testing revealed a trend toward a higher decay rate in SM than in both PM (*p* = 0.06) and LM (*p* = 0.052). **d**. Group mean responses per minute per trial under a PR4 schedule. **e**. The predicted peak response rate in PR (one-way ANOVA, F = 6.25, df = 2,39, *p* < 0.005). Post-hoc tests revealed significant differences between PM and LM (*p* < 0.01) and SM and LM (*p* < 0.01). **f**. Response decay rate in PR (one-way ANOVA, F = 0.62, df = 2,39, *p* = 0.54). #*p* < 0.05 in one-way ANOVA; *p < 0.05, **p < 0.01 in Tukey’s post hoc comparison

To further explore calorie-dependent temporal changes in operant responding within a behavioral session, we analyzed changes in the response rate within a session of the FR5 and PR4 schedules (Fig. 3). Both SM and PM supported a high initial response rate, whilst animals reinforced by LM responded at a significantly lower rate initially. These findings indicate that overall behavioral outputs indicated by total trial number in FR (Fig. 1a) and breakpoint in PR (Fig. 2a) are primarily determined by initial (peak) response rate within a session of FR (Fig. 3a, b) and of PR (Fig. 3d, e), suggesting that reinforcer caloric content affects behavioral output from the very beginning of a behavioral session. Interestingly, we found that within-session response rate decay was dependent specifically on reinforcer sugar content rather than total caloric content in the FR (Fig. 3a, c), but not in the PR schedule (Fig. 3d, f). Given that the FR schedule can index changes in satiation within a session, this finding may suggest that satiation is achieved more rapidly by high-sugar versus high-fat content reinforcers even under isocaloric conditions. In contrast, the overall incentive value of a reinforcer as indicated by the trial number in the time-limited FR schedule and the breakpoint in the PR schedule is determined primarily by total caloric content.

Higher reinforcer caloric content supports faster training in touchscreen ratio schedules.

**Fig. 4:**
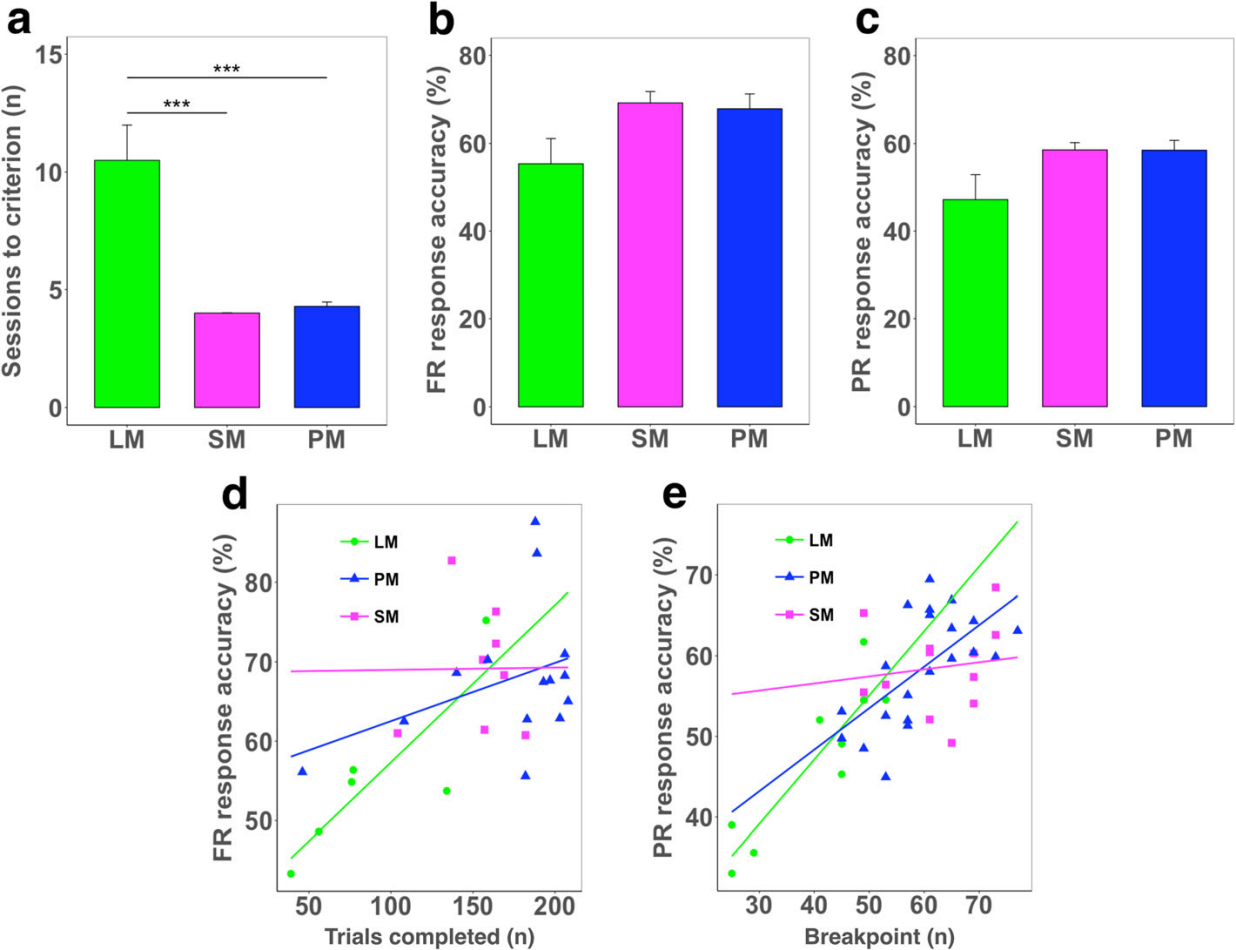
Rate of training and response accuracy in touchscreen ratio schedules **a**. Sessions required to reach criterion (30 FR5 trials completed in 60 min) (one-way ANOVA, F = 24.75, df = 2,12, *p* < 0.0001). **b**. Response accuracy [correct responses × 100 / correct and blank responses (%); one-way ANOVA, F = 3.91, df = 2,10, *p* = 0.056] in the unrestricted FR5 schedule. **c**. Response accuracy in the PR4 schedule [correct responses × 100 / correct and blank responses (%); one-way ANOVA, F = 3.76, df = 2,11, *p* = 0.057]. **d**. Relationship between the number of trials completed and response accuracy in FR (Pearson’s r; whole, *r* = 0.61, *p* = 0.001; LM, *r* = 0.84, *p* = 0.04; SM, *r* = 0.01, *p* = 0.98; PM, *r* = 0.38, *p* = 0.18). **e**. Relationship between perceived incentive value (breakpoint) and response accuracy in PR (whole, *r* = 0.75, *p* < 0.001; LM, *r* = 0.90, *p* = 0.001; SM, *r* = 0.14, *p* = 0.67; PM, *r* = 0.63, *p* = 0.002). *n* = 4–7 per group. LM, low-fat milk; SM, strawberry-flavored milk; PM, plain white milk. ***p < 0.001 by Tukey’s post hoc comparison

As we found that the incentive value of reinforcers depends on total caloric content, we analyzed sessions to criterion (the number of sessions required to the first completion of 30 trials in FR5 within 60 min) in FR training and response accuracy [correct: total (correct plus incorrect) response ratio] in the FR5 and PR4 schedules to see if reinforcer incentive value affects the speed of training (rule acquisition) and response accuracy in the touchscreen apparatus. Sessions to criterion differed significantly between groups according to reinforcer caloric content (Fig. 4a and Additional file 1: Figure S1). Trends toward between-group differences in response accuracy were also detected (*p* = 0.06; Fig. 4b and c) and accuracy scores of individual animals were robustly correlated with the total number of trials completed (Fig. 4d) and with breakpoint (Fig. 4e) in the FR and PR schedules, respectively. Taken together, these analyses suggest that speed and accuracy in operant learning in the touchscreen system may be highly dependent on reinforcer strength which is primarily dependent on caloric content.

### Different isocaloric milk-based reinforcers produce equivalent behavioral performance in two independent laboratories

**Fig. 5:**
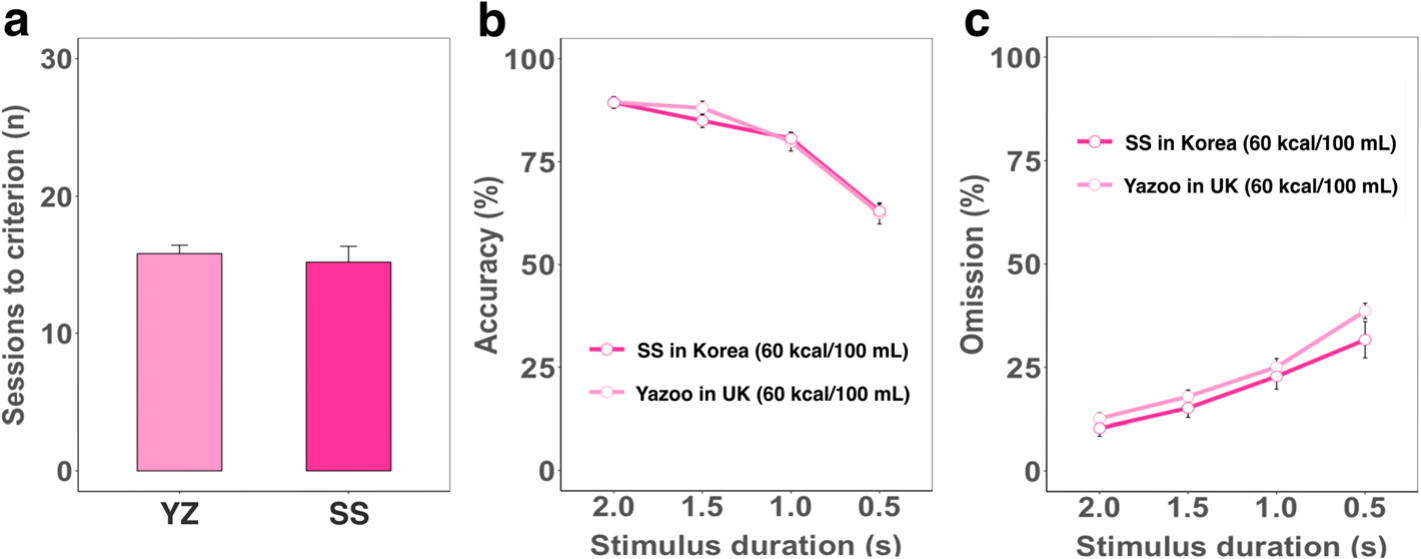
5-CSRTT performance in two independent cohorts reinforced with different isocaloric reward liquids. Behavioral outcomes from two separate cohorts of mice (*n* = 15–16/each cohort) of the same strain (C57BL/6) and age (6 months), assessed by independent labs (in Korea and the UK) with different isocaloric milk products (Additional file 2: Table S1) in the Bussey-Saksida touchscreen apparatus. **a**. Sessions to criterion, the number of sessions required to reach >80% accuracy and <20% omissions in baseline training (one-way ANOVA, F = 0.23, df = 1,30, *p* = 0.636). **b**. Accuracy (% of correct responses out of all responses made; mixed-effect model, main effect of group, F = 0.08, df = 1,29, *p* = 0.779; main effect of stimulus duration, F = 104.99, df = 3,455, *p* < 0.0001). **c**. Omission (% of trials missed out of all trials; main effect of group, F = 1.39, df = 1,29, *p* = 0.249; main effect of stimulus duration, F = 66.08, df = 3,455, *p* < 0.0001). YZ, Yazoo® used in UK cohort; SS, Seoul Strawberry milk® in Korean cohort

To identify whether isocaloric reinforcers indeed produce consistent behavioral outcomes irrespective of other differences in nutritional composition, manufacturer or country of origin, we compared the performance of mice trained in the touchscreen 5-CSRTT run independently by research groups in Korea and the UK using different kinds of isocaloric milk-based products (60 kcal/100 mL): a Seoul Strawberry Milk® (Korea, SS) and Yazoo® (UK; Additional file 2: Table S1). The 5-CSRTT was chosen for this comparison as it is one of the most widely used operant assays for mice [5, 16]. Furthermore, during probe sessions animals experience profound, unpredictable changes in cognitive demand due to variation in stimulus duration, which varies attentional load. These unpredictable challenges provide a stringent test of reproducibility of behavioral performance. Weights of animals during the probe sessions were higher in the UK cohort than in Korean cohort, because food restriction was commenced at older age in the former (Additional file 1: Figure S1b). Despite the difference in average weight between the two cohorts, sessions to criterion (Fig. 5a), accuracy (Fig. 5b), and omission scores (Fig. 5c) were highly comparable between the two cohorts irrespective of stimulus duration challenge.

## Discussion

Previous findings have shown that liquid reinforcers are effective and potent in supporting operant learning in mice [3, 9, 10]. However, the identity of the factor(s) that determine the incentive potential of nutritionally distinct liquid reinforcers remains elusive. Given the regional and national variability in product supplies, this could introduce a significant uncontrolled factor into behavioral studies conducted at different institutions, potentially making comparison between studies difficult and introducing issues of non-reproducibility via increased variability between sites involved in multi-laboratory research collaborations. Therefore, despite the standardized apparatus and software now available for operant behavioral assessment, comparison of study outcomes could be compromised by variability derived from differences in the reinforcer used.

To address this issue and thereby help to improve standardization and replicability of operant performance across laboratories, we compared the reward strength of several milk-based liquid reinforcers that have distinct nutritional properties. Our findings show that reinforcer caloric content, rather than fat or sugar content in isolation, or flavor, is the most influential contributing factor. This suggests that consistency in unit caloric content is the critical component that requires standardization across liquid reinforcers. We also found that higher calorie reinforcers, such as SM and PM not only supported higher responding under FR and PR schedules but also faster training acquisition. This information is useful for investigators when selecting an operant reinforcer in that it will help to minimize training time.

Given the suggested importance of total reinforcer caloric content, we tested this prediction by comparing performance of two independent animal cohorts in two distinct laboratories using the same apparatus and protocols on a challenging behavioral task reinforced with two different but isocaloric milk-based reinforcers. Task performance was highly consistent between the two sites and notably, patterns of performance changes across the various degrees of task difficulty (stimulus duration) were also consistent between the two cohorts. These findings support the importance of reinforcer total caloric content (as opposed to individual factors such as fat or sugar composition) as the factor most in need of standardization to maximize consistency in operant behavioral studies across labs.

Our findings are consistent with the view that satiety is a primary source of food reward, which may serve to enable energy homeostasis [17]. Indeed, when comparing two higher isocaloric reinforcers (SM and PM), no detectable effects of sweetness (sugar) or flavour (strawberry) on overall levels of motivation and speed of training were identified. In fact, a series of previous studies reported that the nutritional (caloric) rather than hedonic (taste) value is a primary component of food-seeking behavior [18–20] and can modify brain reward circuits [21, 22].

Interestingly, the within-session response rate analysis of FR (Fig. 3a,c) showed that PM (high-fat/low-sugar) induced a lower decay rate in responding relative to SM (low-fat/high-sugar). Such a difference in decay rate between PM and SM (Fig. 3c) may indicate differential post-ingestive feedback effects between fat and sugar; a post-ingestive positive feedback effect (conditioned acceptance) exerted by fat and a negative feedback effect (conditioned satiation) of sugar, consistent with previous studies [23–25]. Alternatively, the decay rate may depend on sugar content itself as indicated by the unexpected similarity in decay pattern between LM and PM (Fig. 3a,c). A previous study has shown that high concentrations of carbohydrate exert conditioned satiation whereas low concentrations induce conditioned acceptance [26]. Taken together, our data suggest the possibility that sugar content affects satiation rate while total caloric content primarily governs food-seeking behavior. Thus, it could be speculated that a low-sugar and high-fat reinforcer would likely help keep animals engaged throughout a prolonged task session (e.g., > 60 min), although more data are required [27].

It is notable that such differential effect of high- versus low-sugar on satiation was only observed in the unrestricted FR schedule, but not in the PR schedule. In FR, satiation may be a key factor influencing the decay rate of responding because constant and mild degree of effort is needed to receive a reward in every trial. By contrast, in PR schedule, there should be a trade-off between effort and desire for reward because efforts to get a reward are increasingly required in every subsequent trials. Thus, it is likely that effect of satiation by high-sugar on animal behavior was, if any, little in PR schedule in which the total amount of reward intake is generally small.

Several limitations may concern this study. We only presented the result of sessions to criterion in FR training and response accuracy, which were dependent on motivational levels (Fig. 4), to show the effect of reward strength on learning. Although FR/PR schedules are considered canonical tests for evaluation of motivation and reward strength [14], differential effects of various caloric content on speed of learning would have been better examined by a standard discriminative learning paradigm. In fact, our recent study has shown that reinforcers with different caloric contents (milkshake versus ‘super-saccharin’) induces different speed of learning in visual discrimination learning [9]. Additional tasks such as extinction could be conducted to support our finding as to the relationship between reward strength and learning because reward strength affects resistance to extinction in the operant learning paradigm.

Secondly, taste and intensity of sweetness were not strictly controlled by proper control group. We could not be yet conclusive about how strawberry flavor or sweetness influences animals’ operant behavior or whether these two factors in SM work interactively. Varying the intensity of sugar content and using PM with the same sugar/caloric content with SM would help address this complex issue in the future. Thus, our finding should be interpreted as not that caloric content solely contributes to the reinforcing effect, but that caloric content is one of the most reliable parameters that should be primarily considered for standardised use of liquid reinforcers. Lastly, caution should be made in the interpretation of the regression lines shown in Fig. 4d,e; they might appear to indicate as if motivation-dependent increase in accuracy is more obvious in LM than SM or PM condition. However, there was no animal in LM condition that showed high degree of motivation at all, and thus data from only several animals that had marked very low accuracy upon low level of responding contributed to steeper slope in the regression line of LM condition than of other conditions.

## Conclusions

In conclusion, the unit caloric content is an important element of the incentive value of milk-based products, which can be used as liquid reinforcers for operant behavior experiments. Higher calories are associated with increased levels of motivation in food-seeking behavior and faster training, and isocaloric products generate equivalent task performance. Therefore, reinforcer standardization can be achieved by matching total caloric content across laboratories using (touchscreen) operant equipment despite regional or national differences in reinforcer product supplies.

## Methods

### Animals

For Korean cohort: Male C57BL/6 J mice (Central Lab, Animal Inc., Seoul, Korea) were purchased at 5 weeks of age and housed in groups of 3–4 per cage in a humidity- and temperature-controlled, specific pathogen-free housing room in the Yonsei University College of Medicine Animal Care Facility. All animal experiments were approved (No. 2015–0324) by the Animal Care Committee of Yonsei University College of Medicine with NIH guidelines. Mice had a 1-week acclimatization period prior to commencing procedures. Cages and water bottles were changed once a week. Animals were trained in the touchscreen chamber once a day for 5–6 days a week, during the light phase of the 12 h light-dark cycle (light on at 8:00 am). Food restriction was used to maintain mice at approximately 85% of free-feeding weight throughout the experiment. At the initial stage of food restriction, the daily provision of chow pellets was adjusted to avoid weight loss of more than 5% from the previous day. Weights were measured daily throughout the study. For UK cohort: Male C57BL/6JBabr mice (Babraham Institute, Cambridge, UK; a inbred strain of C57BL/6 J purchased from Charles River UK, Margate, UK) were housed in groups of 2–5 per cage under a 12 h light-dark cycle (light on at 7:00 pm). All testing was conducted during the dark phase. All other procedures such as acclimatization, food restriction, weight control and drinking water access were identical to those used in the Korean cohort. All experiments were conducted in accordance with the United Kingdom Animals (Scientific Procedures) Act (1986).

### Materials

Three different milk products were selected as reinforcers in this study (Table 1); a semi-skimmed low-fat milk (LM; Seoul Low-fat Milk®), a strawberry-flavored milk (SM; Seoul Strawberry Milk®), and a plain white milk (PM; SeoulMilk®), all of which were purchased from SeoulMilk Dairy Cooperative (Seoul, Korea). These milk products were selected with an intent to contrast different factors (Table 1) potentially related to incentive value such as total calories (low versus high), sweetness (sugar content; low versus high), fat content (low versus high), and flavour (strawberry versus plain). For the comparison between the Korean and UK cohorts, Yazoo®, a strawberry milkshake from FrieslandCampina UK (Horsham, UK) and another strawberry flavoured milk product from Korea (Seoul Strawberry Milk®; SS) were used. SS (Additional file 2: Table S1), which is isocaloric to Yazoo®, is a different product to the SM used in the FR and PR experiments reported here (see Table 1).

### Apparatus

All training and testing was conducted in standard Bussey-Saksida mouse touchscreen chambers (Campden Instruments Ltd., Loughborough, UK), which are fully described elsewhere [3, 10]. Briefly, the touchscreen (12.1 in..; resolution 800 × 600) is surrounded by a stainless-steel floor and a trapezoidal reinforced plastic wall. Infrared beams are positioned around the chamber to detect animals’ movement. The front infrared beam is 6 cm away from the screen, and the rear beam is 3 cm away from the magazine (reward tray). Another infrared beam detects head entries to the magazine port. A magazine beam break (via head entry) is used as a signal to initiate trials. The magazine contains a light-emitting diode (LED), which illuminates coincident with reward delivery, which is 20 μL per trial. When mice collect reward, the LED is turned off, and the next trial begins. A black plastic mask is used to partially cover the touchscreen and has a row of 5 square (4 × 4 cm) holes (Campden Instruments Ltd) which are situated at 1.5 cm above the floor. A visual stimulus appeared only in the central response location in the FR and PR tasks, whereas all locations were used in the 5-CSRTT.

### Behavioral task procedures

Habituation was performed for at least 2 days to allow the animals to become accustomed to the chamber and the reward before the behavioral experiment. This step lasted for 20 min and was conducted on 2 consecutive days. We delivered 200 μL of reward to the magazine tray before the beginning of each habituation session. When general movement of mice quantified by front and rear infrared beams indicated no abnormality in locomotor behavior, and consumption of all reward was identified on 2 consecutive days, initial touch training was started on the next day. In this step, mice were trained to associate stimulus offset with reward delivery. There was a limit of 60 min and 30 trials per session, and the stimulus was maintained for 30 s on the screen. When mice touched the stimulus, a tone (1000 ms, 3 kHz) was produced and triple the standard reward volume (60 μL) given. If the stimulus was not touched, the standard reward volume (20 μL) was given upon stimulus offset. Upon reward collection, the LED is turned off in the magazine and followed by an inter-trial interval (ITI) of 5 s. Then a new trial begins. Once mice completed 30 responses within 60 min, they were placed on the FR schedule.

Upon completion of pre-training, animals were trained specifically for FR and PR schedules. Initially, mice were trained to collect a single reward by emitting a single touchscreen response, which is defined as an FR1 schedule. Next, mice were trained stepwise for FR2, FR3, and FR5, as described elsewhere [10, 15]. In FR5, mice should emit 5 operant responses to earn a single reward. A single trial constitutes these 5 responses. Upon successful completion of FR5 training (finishing 30 trials within 60 min), mice underwent an ‘unrestricted’ FR5 session, which had no limitation on the number of trials within a 60 min session. After two sessions of unrestricted FR5, three sessions of a PR4 schedule were conducted. In the PR4 schedule, the required number of responses to receive a single reward was progressively increased on a linear +4 basis (i.e. 1, 5, 9, 13, and so on) in each subsequent trial. Sessions were run for 60 min, but if mice did not touch the stimulus or collect a reward delivered to the magazine within 5 min, the session was terminated. In PR, the breakpoint, total screen touches and blank touches were measured. Breakpoint is defined as the number of target responses emitted by mice in the last trial in which reward was successfully earned within a session. In all touchscreen experiments, the degree of animal handling by experimenters was minimal. Each animal was daily weighed before introduction to the touchscreen chamber. Upon completion of a session, they were removed from chambers, and were given pellets into their cage by an experimenter.

### Comparison of behavioral outcomes from two independent cohorts

The data were collected from two independent studies (unpublished) conducted in Republic of Korea and the UK using the same strain of mice (8–12 weeks old, male C57BL/6, *n* = 15–16 per group). All experimental conditions including the protocol, equipment, and software for task control and data acquisition [5] were identical between the two sites except reinforcers used; either of two isocaloric milk-based beverages, Yazoo milkshake® (UK; 60 kcal/100 mL) or Seoul Strawberry Milk® (Korea; 60 kcal/100 mL; SS; Additional file 2: Table S1) was used. Procedures for the 5-CSRTT are detailed elsewhere [5, 16]. Briefly, animals were trained to respond to a stimulus appearing at one of the five spatial locations on the touchscreen. Upon a correct response, 20 μL of the liquid reinforcer was pumped out into the magazine with no delay. The contrast in whiteness was set at 100% (‘lamp 100’) between the stimulus (visual cue) and background. Following completion of 12-week training, animals (24 weeks old) were challenged with a probe session in which stimuli were presented with a range of durations. We analyzed number of sessions to criterion (the number of training sessions required to reach the criterion of >80% accuracy and <20% omission at a stimulus duration of 2 s), and accuracy (correct responses per total responses made) and omissions (the number of trials in which no response made) in the stimulus duration probe session.

### Statistical analysis

Statistical analyses were conducted using R version 3.3.0 [28]. Figures were constructed using the ggplot2 package in R [29]. Between-group comparisons were analyzed using a linear mixed-effects model or one-way ANOVA with Tukey’s honest significance difference post hoc test. Data from the unrestricted FR5 and PR4 schedules were analyzed based on mean performances across two and three consecutive sessions respectively. The within-session response rate analysis in FR and PR was conducted as fully described elsewhere [9, 30]. Briefly, total response time data (the first to last touchscreen response in a single trial) were converted to rate (responses per minute). Subsequently, these were fitted with the equations, y = b*(x)^2 + *a* for FR and y = *a*^(−b*x) for PR using non-linear least square regression. From these equations, predicted values for peak response rate (*a*) and decay rate (*b*) for individual animals were extracted. 5-CSRTT data were analyzed based on four sessions of the variable stimulus duration probe. Correlations were measured using Pearson’s analysis. The significance level was set at α < 0.05. Data were expressed as mean ± standard error of mean.

## Additional files

Additional file 1: Figure S1. Weights of animals between groups during the tasks. a. Average weight of animals during the unrestricted FR5 and PR4 schedules (one-way ANOVA; FR, F = 1.62, df = 2,11, *p* = 0.24; PR, F = 0.96, df = 2,11, *p* = 0.412). *n* = 4-7 per group. b. Average weight of animals between the UK and Korea during the probe session of 5-CSRTT (t = 5.98, df = 29, *p < 0.001). FR, fixed-ratio; PR, progressive-ratio; 5-CSRTT, 5-choice serial reaction time task; LM, low-fat milk; SM, strawberry-flavored milk; PM, plain white milk; YZ, Yazoo® strawberry milkshake (UK cohort); SS, Seoul Strawberry milk® (Korea cohort). *n* = 15-16 per group. Error bar indicates mean ± SE. **Figure S2.** Accumulated number of sessions required in each stage of FR training. The accumulated number of sessions required to reach the criterion in each stage of FR training revealed significantly reduced speed of learning in LM condition than other conditions (mixed-effect model; main effect of reinforcer, F = 11.3, df = 2,12, *p* = 0.002; main effect of stage, F = 219.4, df = 3,36, *p* < 0.0001; reinforcer by stage interaction, F = 33.2, df = 6,36, *p* < 0.0001). FR, fixed-ratio; LM, low-fat milk; SM, strawberry-flavored milk; PM, plain white milk. *n* = 4-7 per group. Error bar indicates mean ± SE. *p < 0.05, ***p < 0.0001 in simple main effect of reinforcer at each stage. (DOCX 1595 kb)

Additional file 2: Table S1. Comparison between Yazoo® and Seoul Strawberry Milk®. (DOCX 38 kb)

## Abbreviations

5-CSRTT: 5-choice serial reaction time task
ANOVA: analysis of variance
FR: fixed ratio
IR: infrared
ITI: inter-trial interval
LED: light-emitting diode
LM: low-fat milk
PM: plain milk
PR: progressive ratio
SM: strawberry-flavored milk
SS: Seoul Strawberry Milk
