# Supplementary material for: Optimizing reproducibility of operant testing through reinforcer standardization: identification of key nutritional constituents determining reward strength in touchscreens
Source: Mol Brain. 2017 Jul 17;10:31. doi: 10.1186/s13041-017-0312-0 (PMC5512767; doi:10.1186/s13041-017-0312-0)

**Supplementary Fig. 1. Weights of animals between groups during the tasks**

**a.** Average weight of animals during the unrestricted FR5 and PR4 schedules (one-way ANOVA; FR, F = 1.62, df = 2,11, p = 0.24; PR, F = 0.96, df = 2,11, p = 0.412). n = 4-7 per group. **b.** Average weight of animals between the UK and Korea during the probe session of 5-CSRTT (t = 5.98, df = 29, *p < 0.001). FR, fixed-ratio; PR, progressive-ratio; 5-CSRTT, 5-choice serial reaction time task; LM, low-fat milk; SM, strawberry-flavored milk; PM, plain white milk; YZ, Yazoo® strawberry milkshake (UK cohort); SS, Seoul Strawberry milk® (Korea cohort). n = 15-16 per group. Error bar indicates mean ± SE.

**b**

**a**


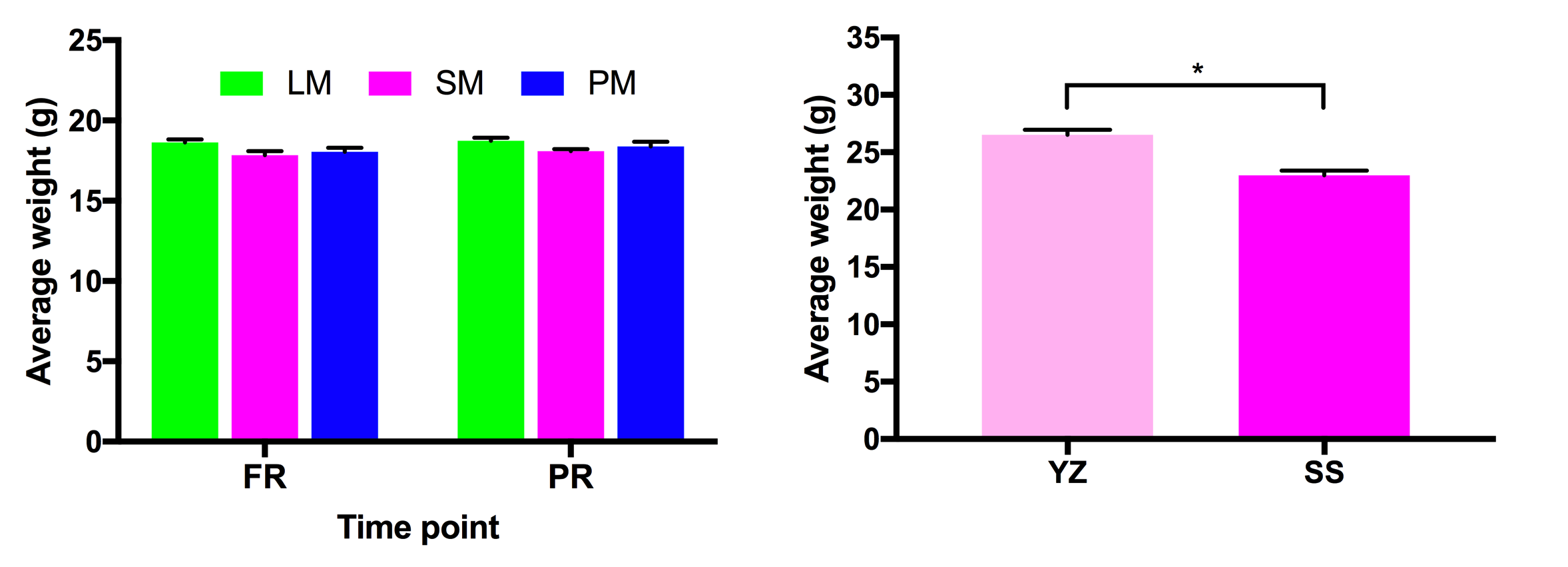


**Supplementary Fig. 2. Accumulated number of sessions required in each stage of FR training.**

The accumulated number of sessions required to reach the criterion in each stage of FR training revealed significantly reduced speed of learning in LM condition than other conditions (mixed-effect model; main effect of reinforcer, F = 11.3, df = 2,12, p = 0.002; main effect of stage, F = 219.4, df = 3,36, p < 0.0001; reinforcer by stage interaction, F = 33.2, df = 6,36, p < 0.0001). FR, fixed-ratio; LM, low-fat milk; SM, strawberry-flavored milk; PM, plain white milk. n = 4-7 per group. Error bar indicates mean ± SE. *p < 0.05, ***p < 0.0001 in simple main effect of reinforcer at each stage.


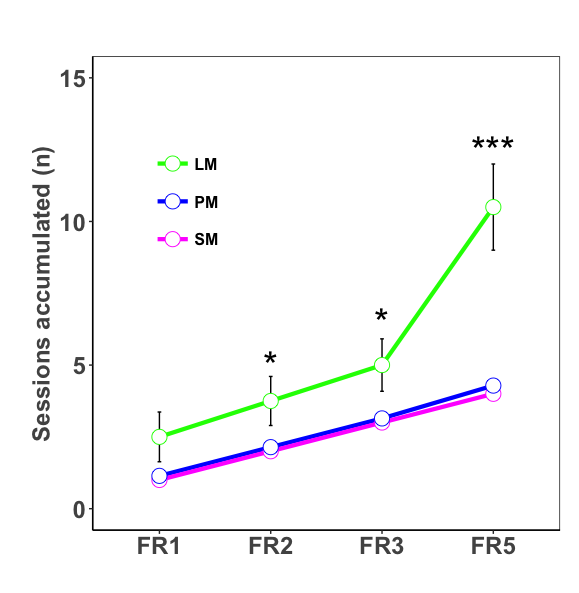

Supplement: Supplementary file 1 — Weights of animals between groups during the tasks. a. Average weight of animals during the unrestricted FR5 and PR4 schedules (one-way ANOVA; FR, F = 1.62, df = 2,11, p = 0.24; PR, F = 0.96, df = 2,11, p = 0.412). n = 4-7 per group. b. Average weight of animals between the UK and Korea during the probe session of 5-CSRTT (t = 5.98, df = 29, *p < 0.001). FR, fixed-ratio; PR, progressive-ratio; 5-CSRTT, 5-choice serial reaction time task; LM, low-fat milk; SM, strawberry-flavored milk; PM, plain white milk; YZ, Yazoo® strawberry milkshake (UK cohort); SS, Seoul Strawberry milk® (Korea cohort). n = 15-16 per group. Error bar indicates mean ± SE. Figure S2. Accumulated number of sessions required in each stage of FR training. The accumulated number of sessions required to reach the criterion in each stage of FR training revealed significantly reduced speed of learning in LM condition than other conditions (mixed-effect model; main effect of reinforcer, F = 11.3, df = 2,12, p = 0.002; main effect of stage, F = 219.4, df = 3,36, p < 0.0001; reinforcer by stage interaction, F = 33.2, df = 6,36, p < 0.0001). FR, fixed-ratio; LM, low-fat milk; SM, strawberry-flavored milk; PM, plain white milk. n = 4-7 per group. Error bar indicates mean ± SE. *p < 0.05, ***p < 0.0001 in simple main effect of reinforcer at each stage. (DOCX 1595 kb) [file 13041_2017_312_MOESM1_ESM.docx]
