# Supplementary material for: Optimizing reproducibility of operant testing through reinforcer standardization: identification of key nutritional constituents determining reward strength in touchscreens
Source: Mol Brain. 2017 Jul 17;10:31. doi: 10.1186/s13041-017-0312-0 (PMC5512767; doi:10.1186/s13041-017-0312-0)
Supplement: Supplementary file 2 — Comparison between Yazoo® and Seoul Strawberry Milk®. (DOCX 38 kb) [file 13041_2017_312_MOESM2_ESM.docx]

**Supplementary Table 1. Comparison between Yazoo® and Seoul Strawberry Milk®**

| **per 100 mL** | **Yazoo**  **Milkshake®**  **(UK)** | **Seoul**  **Strawberry Milk® (SS; Korea)** |
| --- | --- | --- |
| **Calories** (kcal) | 60 | 60 |
| **Total Carbohydrates**(g) | 9.6 | 9.5 |
| Sugars (g) | 9.6 | 9 |
| **Protein** (g) | 3 | 2.5 |
| **Total Fat** (g) | 1.2 | 1.6 |
| Saturated Fat (g) | 0.9 | 1 |
| **Salt** (g) | 0.1 | 0.04 |
